# Active Humoral Response Reverts Tumorigenicity through Disruption of Key Signaling Pathway

**DOI:** 10.3390/vaccines10020163

**Published:** 2022-01-21

**Authors:** Tracer Yong, Ko-Keng Chang, Yi-Sheng Wang, Che Ma

**Affiliations:** 1 Genomic Research Center, Academia Sinica, Taipei 11529, Taiwan; tracer.yong@ki.se (T.Y.); fticr@gate.sinica.edu.tw (K.-K.C.); wer@gate.sinica.edu.tw (Y.-S.W.); 2 Chemistry Department, National Taiwan University, Taipei 10617, Taiwan

**Keywords:** immunotherapy, peptide cancer vaccine, B-cell cancer vaccine, CRM197, IL-17RB, MALDI-TOF-MS, hapten density, 4T1

## Abstract

Immune checkpoint inhibitors such as monoclonal antibodies (mAbs) are amongst the most important breakthroughs in cancer therapeutics. However, high cost and short acting time limits its affordability and clinical application. Therefore, an economical and durable alternative is urgently needed. Previously, we identified an IL-17RB targeting mAb which intercepts IL-17B/IL-17RB signal transduction and suppresses tumorigenesis in many types of cancer. We reason that active immunity against the antigenic epitope of IL-17RB can reproduce the anti-cancer effect of mAbs with better sustainability. Here, we present a cancer vaccine composed of multiple synthesized epitope peptides chemically conjugated onto CRM197, a highly immunogenic carrier protein. Combining mass spectrometry with immunoassay, we standardized hapten density determination and optimized vaccine design. Furthermore, orthotopically transplanted syngeneic mouse tumor 4T1 showed that administration of this vaccine therapeutically mitigates primary cancer growth as well as distance metastasis. In conclusion, we demonstrate preparation, characterization and pre-clinical application of a novel peptide cancer vaccine.

## 1. Introduction

It is well known that paracrine/autocrine cytokine signaling pathways are taken advantage of by cancer cells to promote disease progression, immune evasion and metastasis [1,2,3]. Immune checkpoint inhibitors, including monoclonal antibodies (mAbs), have been shown effective in disrupting extracellular signaling and successfully applied in clinical use [4,5,6]. However, their short half-life nature dictates a high cost for prolonged usage [7] which, in extreme cases, can reach over USD 750,000 annually per patient [8]. This largely limits its affordability and application especially against minimal residual disease or extravasated circulating tumor cells and it may be years before they proceed to an eventual relapse [9]. Thus, a sustainable and cost-effective alternative to therapeutic mAb is needed.

Active humoral response through vaccination has successfully eradicated some of the most notorious infectious diseases [10], and its role as a cancer therapeutic is emerging [7,11,12,13]. Several lines of research agree that peptide-based vaccine is a valid strategy inducing specific immune response against tumor associated antigens [7,12]. However, short peptides with suboptimal immunogenicity often induce immunotolerance towards these epitopes and even enhance tumor growth [14,15,16]. Thus, different strategies such as conjugation, adjuvant addition or both have been applied in the hope of promoting immunogenicity of the peptide vaccine. For example, a potent and specific IgG response against mouse programmed cell death protein 1 (mPD1) was achieved through conjugating mPD1 mimotope onto cross reactive material 197 (CRM197), a non-toxic, highly immunogenic mutant of diphtheria toxoid (DT), which has been widely applied to vaccine preparation [17] and has successfully demonstrated its anti-tumor effects [18]. Despite this, only the prophylactic use of the vaccine was successful in most pre-clinical studies so far [18,19,20,21], suggesting further optimization is needed for active humoral response to become a cancer therapy.

Previously, we reported the findings that interleukin 17 receptor B (IL-17RB) signaling contributes to cancer survival, proliferation and metastasis upon binding to its cognate ligand IL-17B [22,23]. Utilizing qRT-PCR, immunoblotting and RNA interference, we revealed its cellular mechanism to be upregulating pro-inflammatory cytokine expression in an ERK1/2 dependent fashion in multiple cancer cell lines [24]. We also reported isolation of a monoclonal antibody, known as D9, disrupting IL-17B:IL-17RB binding and conferring therapeutic effects in a patient-derived xenograft animal model. More recently, we defined the linear epitope of IL-17RB, hereafter named IL-17RB inactivation site (IRIS), through X-ray crystallography [25]. This evidence poises IRIS as a potential target for novel anti-cancer therapeutics.

In this research, we demonstrate the development of a peptide-based vaccine composed of multiple IRIS peptides chemically conjugated onto CRM197. To characterize the vaccine in detail, we utilized matrix-assisted laser desorption/ionization time-of-flight mass spectrometry (MALDI-TOF-MS) to enable hapten density estimation as a crucial step in quality control and immunogenicity optimization. ELISA and flow cytometry verified a positive correlation between immunogenicity and hapten density of our vaccine, as well as its potency and specificity in the animal model. Finally, we show that therapeutic use of our vaccine suppresses primary tumor growth and distance metastasis in the syngeneic mouse model through disruption of IL-17RB downstream signaling pathway. 

## 2. Materials and Methods

### 2.1. CRM197 Stock Preparation

Lyophilized CRM197, purchased from Reagent Proteins (San Diego, CA 92121, USA), was dissolved in pre-chilled deionized water, buffer-exchanged for pre-chilled PBS by three successive rounds of 30 kD molecular weight cut-off Amicon^®^ Ultra Centrifugal Filter unit (Millipore, Burlington, MA, USA), and it was then adjusted to the concentration at 1 mg/mL. All procedures were carried out at 4 °C or on ice.

### 2.2. SBAP Activation

Succinimidyl 3-(bromoacetamido) propionate (SBAP) activation was carried out with the concentration of 0.38 mg of CRM197/mL at the molar ratio of CRM197:SBAP = 1:240; in pH = 8, 0.1 M of sodium phosphate buffer. The reaction was incubated for 2 h at 4 °C in the dark with slight stirring, before being stopped by three successive clean-up rounds using a 30 kD MWCO Amicon^®^ Ultra Centrifugal Filter unit (Millipore, Burlington, MA, USA) with 0.1 M of sodium phosphate buffer with sodium hydroxide at pH = 9 in preparation for the conjugation step. All procedures were carried out at 4 °C or on ice.

### 2.3. Peptide Conjugation

The reaction conditions for peptide conjugation were as follows: 0.15 mg of activated CRM197/mL with the molar ratio of CRM197:peptide = 1:30, 1:60 or 1:90, in pH = 9, 0.1 M sodium phosphate buffer. The reaction was allowed to proceed for 20 h at 4 °C in the dark with slight stirring, before being stopped by adding 10 mg of L-Cystine directly into the reaction regardless of reaction volume (typically 5~10 mL), followed by a 15-min incubation. Next, the reaction was cleaned-up with three successive rounds using 30 kD MWCO Amicon^®^ Ultra Centrifugal Filter unit (Millipore, Burlington, MA, USA) with deionized water. A small fraction of samples were analyzed by MALDI-TOF-MS. After that, the buffer was exchanged for PBS with another three successive rounds of buffer exchange by a 30 kD MWCO Amicon Ultra Centrifugal Filter unit. Finally, the conjugated sample was passed through a Millex-GV 0.22 µm filter unit before use. All procedures were carried out at 4 °C or on ice.

### 2.4. Mass Spectrometry & Data Analysis

Mass spectrometry analysis of the carrier protein and conjugated vaccine was done by a linear MALDI-TOF mass spectrometer that was designed and built in-house. It has a flight path of 0.45 m, which is optimized based on a the comprehensive theoretical model [26] to provide resolution and sensitivity that is superior to commercial instruments for analyses of macromolecules. 

The carrier protein and conjugated vaccine was dissolved in water with a concentration of 1 mg/mL. Equal volumes of sample solution and matrix solution (α-Cyano-4-hydroxycinnamic acid, saturated in 50% acetonitrile (ACN) and 0.1% trifluoroacetic acid (TFA) was added) were mixed on a stainless-steel sample plate and dried under a vacuum. Mass spectra were acquired with an average of 100 laser events for carrier protein analysis and 2500 laser events for conjugated vaccine analysis due to low signal intensity. Bovine serum albumin (BSA) protein and its multiply-charged ions, covering from ~13 to ~66 kD, was used for mass calibration. Spectra were preprocessed with Savitzky–Golay smoothing to reduce noise and the sensitive nonlinear iterative peak (SNIP) method [27] was used for baseline subtraction. Series of doubly charged ion peaks were fitted (deconvolute) using the Gaussian peak function for further hapten density estimation.

### 2.5. Immunization, Serum Acquisition & ELISA

During immunization, each female BALB/c mice was injected with either PBS alone, CRM197 plus 50 µg alum adjuvant (Alhydrogel^®^ CRODA, Frederikssund, Denmark) or C:IRIS vaccine plus alum adjuvant, mixed well in 100 µL of PBS. For serum acquisition, whole blood from mice was obtained through a submandibular vein puncture and centrifuged at 13,200 RPM for 2 min. The supernatant was taken for another centrifugation at 13,200 RPM for 2 min, to remove residual blood cells and clots before being stored at −20 °C. 

One day before assaying, the 96-well ELISA plate was coated with 50 µL of purified recombinant human or mouse IL-17RB ectodomain, diluted to 5 µg/mL in PBS at 4 °C overnight. Then, the plate was blocked with 1% BSA before being incubated with serially diluted mice sera. The plate was then incubated with anti-mouse IgG (H+L) conjugated with horseradish peroxidase (Jackson ImmunoResearch, West Grove, PA, USA, CB7 4EX UK, Code: 115-035-003). All steps are separated with 3 times the amount of PBS with 0.1% Tween-20 (PBST) wash for 5 min each After that, each well was allowed to react with TMB for 2 min before being stopped with 1 M H_2_SO_4_ for the optical density measurement of 450 nm.

### 2.6. IL-17RB-FL Overexpression and Flow Cytometry

HEK293T cells were maintained in Dulbecco’s Modified Eagle medium (DMEM) supplemented with 10% fetal bovine serum (FBS) and antibiotics/antimycotics. Transfection was done using Lipofectamine^TM^ 3000 Transfection Reagent (ThermoFisher, Waltham, MA, USA, L3000001) following its protocol. A total of 24 h after transfection, cells were detached by Accutase^®^ (San Diego, CA, USA), washed (500× *g* spin-down followed by flow buffer resuspension, repeated once) and then resuspended in a pre-chilled flow buffer (5 mM EDTA, 0.1% FBS in PBS). Primary and secondary staining was carried out on ice with 10^5^ of cells resuspended in mice serum diluted 100 times or anti-mIgG-FITC diluted 200 times using a flow buffer for 1 h. After the final wash, cells were resuspended in 300 µL of flow buffer, forced through a cell strainer into Falcon^®^ (MilliporeSigma, Burlington, VT, USA) tubes before being loaded onto flow cytometry (BD FACSCanto^TM^) for detection.

### 2.7. Tumour Transplantation, In Vivo Imaging, Endpoint and Immunohistochemistry (IHC)

Two weeks before tumor transplantation, cryopreserved GL-4T1, a derivative of mouse breast cancer cell line 4T1, stably expressing GFP and luciferase as described and kindly provided by Dr. Michael Hsiao [23], was revived and allowed to grow and maintain in complete culture media (RPMI1640 supplemented with 10% FBS and antibiotics/antimycotics). Transduction status of revived cells were confirmed by fluorescence microscopy and flow cytometry to make sure the majority (>90%) of the cells remain GFP positive. On the day of transplantation, GL-4T1 cells were detached by 0.05% trypsin-EDTA, washed with PBS before being resuspended in Matrigel^®^ (Corning^®^, New York, NY, USA) to 50,000 cells/mL. Resuspended GL-4T1 were then orthotopically injected into each mouse’s 4th mammary fat pad, with 20 µL each. Transplanted mice were imaged for bioluminescence, described below, on the next day to confirm successful transplantation before the first vaccination.

D-luciferin (BIOSYNTH^®^, Staad, Switzerland) was resuspended in sterile PBS before being aliquoted and stored at −80 °C and thawed on ice immediately before use. For in vivo imaging of the primary tumor, mice were anaesthetized by isoflurane, intra-peritoneally given PBS diluted D-luciferin and imaged with the in vivo imaging system (IVIS^®^, PerkinElmer^®^, Waltham, MA, USA).

To harvest the syngeneic tumor and lungs, mice were first put to sleep under general anesthesia induced by Isoflurane. Next, mice were treated with a cardiac puncture for its endpoint serum before being euthanized by cervical dislocation. The tumor and lungs were then harvested and preserved in ice cold PBS. Each tumor was blotted dry on a clean hand towel, then weighted and fixed in 10% formalin for paraffin embedding and sectioning. The freshly harvested lung tissue was dipped in PBS diluted D-luciferin before being imaged by in vivo imaging system (IVIS^®^, PerkinElmer^®^). Each sample was imaged twice, for 30 s each, consecutively. Raw images were then processed by Living Image Software (PerkinElmer^®^) or ImageJ.

For immunohistochemistry, fixed tumor samples were paraffin embedded, sectioned before staining with anti-ERK1/2 antibody (GeneTex, Hsinchu, Taiwan, Cat No. GTX59618, 1:20 dilution). Samples were counterstained with Hematoxylin for further analysis. 

## 3. Results

### 3.1. IL-17RB Inactivation Site (IRIS) and B Cell Epitope Prediction

**Figure 1:**
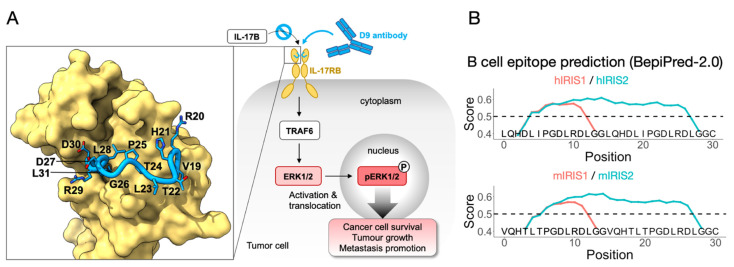
Schematic view of IRIS identification and B-cell epitope prediction. (**A**) Briefly, the D9 monoclonal antibody binding to IL-17RB abrogates IL-17B/IL-17RB downstream signaling and reverts tumor malignancy. Using X-ray crystallography, we previously determined the IL-17RB epitope recognized by D9, namely IRIS, which is shown as a blue loop with side chains in sticks, the rest of the IL-17RB structure is on the yellow surface. (**B**) IRIS1/2 designs using either a human (upper panel) or mouse (lower panel) sequence are subjected to B-cell epitope prediction (BepiPred2.0). The prediction score was set to default (0.5), which is indicated by horizontal dash line.

Our previous work identified IRIS as a working target for monoclonal antibodies to suppress tumor growth and migration (Figure 1A) [25]. We hypothesize that an IRIS peptide vaccine may induce an immune response that will serve to provide a persistent tumor-suppressing environment. Our first step in achieving this was to design the peptide sequence for carrier protein conjugation. IRIS is a 13-residue-long linear epitope located at position 19–31 of bovine IL-17RB (UniProt accession number: A3KN55), which was used in crystal structure determination (Table 1, ID1). The homologous IRIS sequences in mice (mIRIS) and humans (hIRIS) were aligned in the same range, 19–31 (UniProt accession number: Q9JIP3, Q9NRM6) (Table 1, ID2, 3), revealing a 92% and a 77% similarity with bovine IRIS, respectively, suggesting that IRIS is highly conserved across species, especially towards its C-terminus. The sequence differences include V19 (changed to L19 in hIRIS), R20 (changed to Q20 in both mIRIS and hIRIS), T22 (changed to D22 in hIRIS) and T24 (changed to I24 in hIRIS). By checking the atomic structure, none of these residues are involved in direct contact with the D9 antibody except a hydrogen bonding by R20, which could be similarly achieved by Gln in m- or hIRIS [25]. In short, we identified mIRIS and hIRIS for subsequent vaccine preparation.

We then modified the mouse and human IRIS sequence (Table 1, ID2, 3) for subsequent experiments in this research. Specifically acetylation (Ac), which is fused to the N terminus for longer serum half-life [28], and a GGC (Gly-Gly-Cys) peptide, which is fused to the C terminal (Table 1, ID4, 5) to enable conjugation. In the hope of eliciting a strong immune response, we came up with an IRIS2 design with a doubled IRIS epitope load, separated by a GG linker, for mouse and human homologues (Table 1, ID6, 7). We subjected these peptide sequences to BepiPred-2.0, a B-cell epitope prediction algorithm based on annotated antibody-antigen interaction [29], which indicated that both mIRIS1/2 and hIRIS1/2 contain B-cell epitopes (Figure 1B). For h/mIRIS1 design (red curve, Figure 1B), B-cell epitopes were predicted to be located toward their C termini where the IRIS sequence is most conserved, suggesting that h/mIRIS may elicit an antibody response highly cross-reactive with each other. Our preliminary data verified this cross-reactivity, which made us more confident of the algorithm. As for the IRIS2 design, albeit having doubled epitope load, the GG linkers between two IRIS domains, in both human and mouse sequences, are included in the predicted epitopes, which potentially creates novel epitopes that are not specific to IRIS. Therefore, vaccines prepared with IRIS1 or IRIS2 may have their own superiority. In short, we invented two ways of incorporating IRIS in a peptide-based vaccine, and we also utilized a validated B-cell prediction algorithm to predict their immunogenicity.

### 3.2. Vaccine Preparation and Characterization

**Figure 2:**
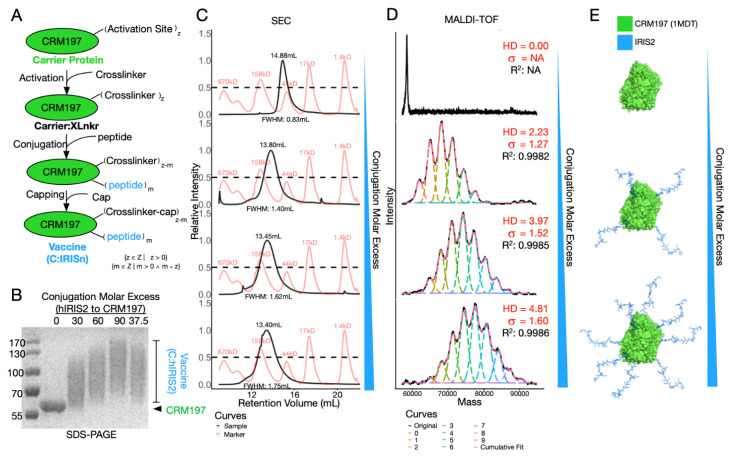
Vaccine preparation and characterization. (**A**) Brief graphical representation of vaccine preparation. (**B**) SDS-PAGE showing vaccine prepared at different peptide:protein molar excess as indicated on top of the image. (**C**,**D**) Vaccine conjugated with increasing peptide:protein molar excess was characterized by SEC and MALDI-TOF-MS. (**C**) CRM197 (top) and C:IRIS samples (2–4 row) are illustrated with black curves whereas standard peaks are shown in red (from the right: 1.4 kD, 17 kD, 44 kD, 158 kD, 670 kD). Retention volume and full width at half maximum (FWHM) for each sample is on the top and bottom of the peak. (**D**) Deconvoluted MALDI mass spectra of the same sample as in panel C, original spectra were shown with a black line, while deconvoluted conjugation species and their cumulative fitted curve are illustrated as dash lines in different colors. Hapten density (HD), standard deviation (dispersity), together with R^2^ of fitted curve are given in the corresponding panel. (**E**) Schematic of the relationship between conjugation peptide molar excess and peptide load.

In order to maximize immunogenicity of IRIS peptide vaccine, we utilized CRM197 as a carrier protein. To facilitate the conjugation, we chose succinimidyl 3-(bromoacetamido) propionate (SBAP) as the crosslinker to bridge primary amines and reduced sulfhydryl groups that are exclusively found on Lysine residues of CRM197 and Cysteine residues of IRIS peptide, respectively. In the first step, around 20 solvent accessible Lysine residues (out of 39 in total) per CRM197 react with excessive SBAP to make activated carrier protein (Carrier:XLnkr, Figure 2A). Then, the IRIS peptide was added to the reaction. During this step, peptide molar excess over CRM197 can be adjusted to achieve differentially conjugated vaccines. Finally, excessive L-Cysteine was added to cap unreacted sites, yielding the final product (C:IRIS). 

Aiming to investigate bioactivity of differentially conjugated vaccines, we applied SDS-PAGE, size exclusion chromatography (SEC) and MALDI-TOF-MS to chemically characterize our conjugates (Figure 2B–D). Upon SDS-PAGE, molecular weight increase corresponded to conjugation molar excess of IRIS over CRM197, ranging from 30:1 to 90:1 (Figure 2B). Of note, C:IRIS displayed wide smeared bands, roughly encompassing 60 to 130 kD (Figure 2B). This suggested the conjugate comprises of multiple conjugation statuses (Figure 2E), which could not be resolved by SDS-PAGE. We then tested differently conjugated C:IRIS with SEC (Figure 2C) and found the retention volume of the single main peak in each run consistently correlated with the carrier-to-peptide ratio, shifting from 14.9 mL to 13.4 mL, suggesting the conjugate is stable and unaggregated in solution (Figure 2C). The shape and width of each SEC peak also underwent subtle changes. While conjugated samples (row 2–4, Figure 2C) exhibited symmetric SEC peaks, a right-skewed curve was observed for the unconjugated sample (top row, Figure 2C), and the peak width, represented by full width at half maximum (FWHM, Figure 2C) of a SEC peak, monotonically increased along with the molar excess, strongly suggesting a decrease in homogeneity. Altogether, conjugation via SBAP has changed not only the molecular weight, but also the composition of the sample, more specifically, pure unconjugated protein may become a mixture of conjugates loaded with different amounts of IRIS peptides. 

To further characterize the conjugation, we utilized the state-of-the-art MALDI-TOF-MS to inspect the molecular weight of each sample (Figure 2D). The mass spectrum of C:IRIS vaccine under different conjugation conditions clearly revealed a series of peaks corresponding to CRM197 loaded with different amounts of IRIS peptides, gradually shifting towards higher *m/z* value as the peptide excess increases (Figure 2D,E). We performed mass spectra deconvolution to recover abundance distributions of each C:IRIS species which enabled a detailed comparison of the conjugation degree between samples. Among all deconvoluted spectra, the adjusted R-square values for cumulative fit were all greater than 0.99, giving confidence to the analysis. With estimated abundance of differentially conjugated species, we define the average hapten density (HD) by the average weight of peak areas and peptide loads as following:

(1) $$\mathrm{HD}=\frac{\Sigma (VUC\;*\;PL)}{\Sigma VUC}$$

where *VUC* refers to peak area under curve for each conjugated species and *PL* denotes peptide load corresponding to predicted *m*/*z* value. As expected, HD value shows strong correlation with increased peptide molar excess during conjugation (Figure 2D,E). Furthermore, these data enable standard deviation (σ, Figure 2D) estimation of HD’s dispersion, which is another essential feature in characterizing conjugates/polymers [30]. In short, we demonstrated HD estimation as a crucial step for C:IRIS characterization, which enables subsequent immunogenicity testing as well as batch-to-batch quality control.

### 3.3. C:IRIS Induces Potent and Specific Immune Response In Vivo with Higher HD

**Figure 3:**
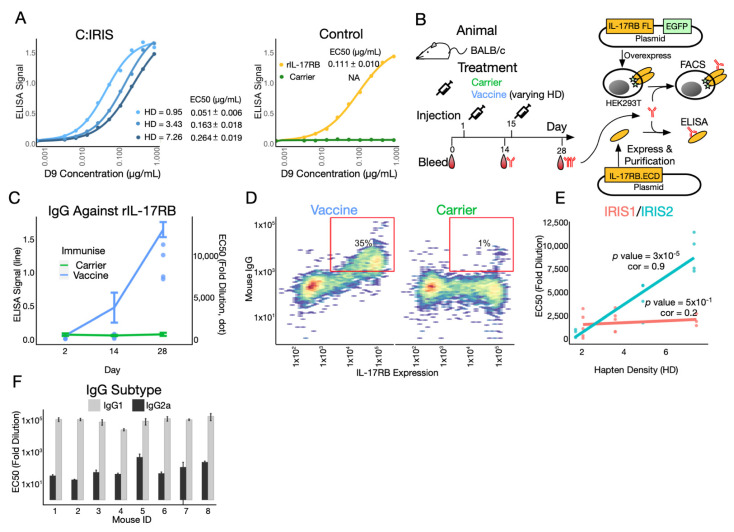
C:IRIS presents natural epitope and induces potent and specific immune response. (**A**) Binding pattern of serial diluted D9 antibody to C:IRIS (Conjugate) and control groups (Control) measured by ELISA. EC50 estimation of D9 antibody binding to different coated antigens is shown in table to the right (**B**) Schematic of the study. C:IRIS (Vaccine) or CRM197 (Carrier) was subcutaneously given to mice at day 1 and 15. Antiserum was collected and tested by ELISA and FACS using purified recombinant IL-17RB ectodomain (IL-17RB.ECD) and IL-17RB overexpressing cells, respectively. (**C**) Day 0, 14 and 28 antiserum from Vaccine- or Carrier-immunized mice were tested for their IgG response against recombinant IL-17RB ectodomain coated ELISA plate. ELISA signal (OD450) from antiserum diluted 810 times is shown as lines and error bars, whereas half maximal effective concentrations (EC50) are shown in dots. (**D**) Using flow cytometry, the IgG response of mice immunized with Carrier or Vaccine is measured by their binding capacity to IL-17RB overexpressing cells from panel B. (**E**) EC50 (fold dilution) of antiserum from mice immunized with C:IRIS prepared with IRIS1 or IRIS2 at different hapten densities (HD). Linear regression is fitted in each panel along with statistical analysis. (**F**) Comparison of EC50 on IgG subtypes (IgG1 and IgG2a) in C:IRIS vaccinated mice.

In order to elicit an immune response specifically targeting the IRIS domain on IL-17RB, it is essential that the C:IRIS conjugate presents native epitope as targeted by D9, the monoclonal antibody targeting IL-17RB. With the ELISA plate coated with C:IRIS with different HD, we found that the D9 antibody binds to C:IRIS and recombinant IL-17RB.ECD, but not to CRM197 (Figure 3A), suggesting that conjugated IRIS peptides do recapitulate native epitope. Surprisingly, we observed a decrease in binding avidity at higher HD (Figure 3A). This is likely due to overcrowded peptides competing for binding on a relatively limited space around C:IRIS and indicates that there might be a trade-off between peptide load and antibody recognition. 

Next, we set out to characterize immunogenicity of C:IRIS in the animal model (Figure 3B). The IgG response towards recombinant IL-17RB ectodomain (rIL-17RB) was not detected in naïve mice serum, nor anti-serum from mice immunized with CRM197 after one or two doses of the injection (Figure 3C). In contrast, mice injected with C:IRIS and alum, the most widely used vaccine adjuvant to enhance immunogenicity, generated minor IgG response against rIL-17RB after 2 weeks, with EC50 ranging between 57~616 fold dilution. This response was substantially increased after a booster of C:IRIS with alum adjuvant, with EC50 reached over 7000 fold dilution for all four of the tested mice, two of which reached over 10,000 fold (Figure 3C). To further verify the immune response being also specific to full length IL-17RB (IL-17RB FL) as presented on the cell surface, we used flow cytometry to test IgG specific binding against HEK293T cells overexpressed with IL-17RB FL fused with an EGFP (Figure 3B). An obvious positive correlation was observed between IL-17RB FL expression, as indicated by EGFP signal, and IgG binding in C:IRIS, but not CRM197-vaccinated mice (Figure 3D). This resulted in a significantly higher frequency of EGFP-Mouse IgG double positive cells in C:IRIS group comparing to CRM197 (35% vs. 1%, Figure 3D), suggesting the presence of anti-IL-17RB IgG after vaccination. Taken together, C:IRIS conjugate can generate a potent and specific humoral response against IL-17RB in vivo.

We next compared immunogenicity of C:IRIS with a different HD and peptide design. For IRIS1 design, three groups of mice (*n* = 5, 4 and 3) were immunized with conjugates with HD at 2.09, 3.63 and 7.51, respectively. A very weak correlation was observed between the HD and immune response (EC50), with the correlation coefficient at 0.2 (*p* = 0.51, Figure 3E). On the other hand, immunogenicity clearly had a better correlation with HD when the epitope load on each peptide was doubled (IRIS2, Figure 3E). Within a comparable HD range (1.73, 4.95 and 7.40, *n* = 3, 3 and 4), the correlation coefficient reached as high as 0.9, and the positive association was confirmed with linear regression (*p* = 3 × 10^−5^, Figure 3E), indicating that the peptide number is a major determinant for immunogenicity over antibody avidity. This is surprising as an earlier result (Figure 3A) suggested a trade-off between HD and immunogenicity. Additionally, ELISA confirmed a humoral response with dominated IgG1 over IgG2a subtype (Figure 3F), consistent with current knowledge that co-administration of alum adjuvant polarizes a Th2 immune response [31]. Collectively, we have shown that immunogenicity of our C:IRIS conjugate vaccine is Th2 polarized and is the strongest when using IRIS2 design with a higher HD value.

### 3.4. C:IRIS Suppresses Cancer Growth & Migration Therapeutically

**Figure 4:**
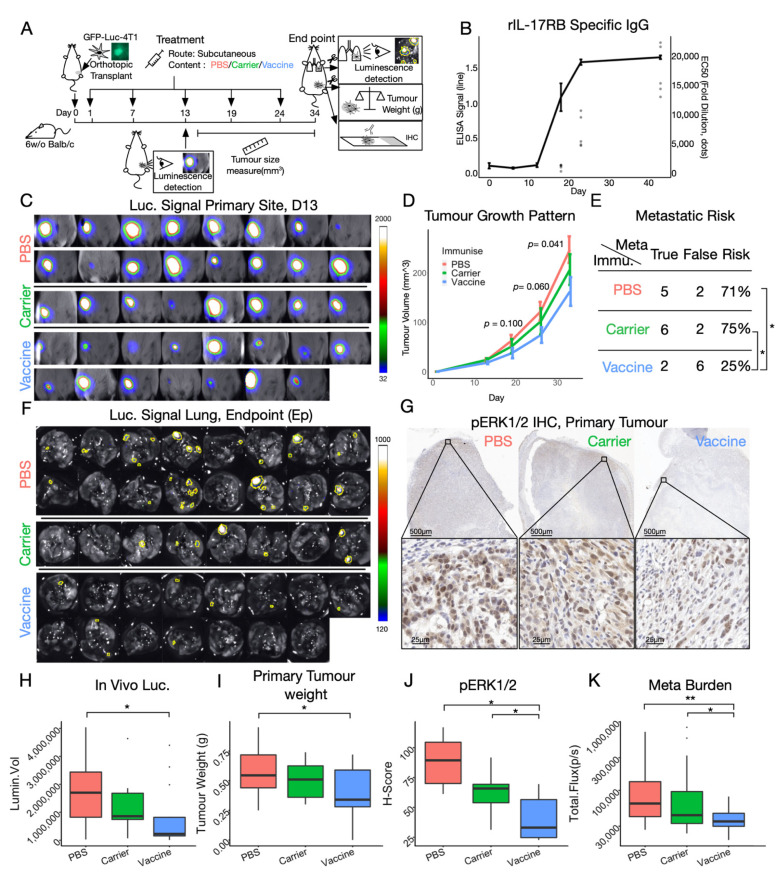
Therapeutic use of C:IRIS vaccine in syngeneic mouse model. (**A**) Schematics of therapeutic animal study design, schedule and data collection. Mice were separated into three treatment groups: PBS, Carrier (CRM197) and Vaccine (C:IRIS). (**B**) Immunogenicity of therapeutic vaccination scheme. BALB/c mice (*n* = 5) were given C:IRIS vaccine as shown in panel A without tumor transplantation and were bled periodically to monitor their immune response. IL-17RB specific IgG response of diluted serum (625×) is shown in line and EC50 as dots. (**C**) Luminescence signal from primary tumors 13 days after transplantation. (**D**) Tumor growing trend in size by treatment is illustrated in different colors. A T test was done between PBS and C:IRIS vaccine (Vaccine) groups in last three observation and p value is shown on top of each observation. Error bar: mean ± SE. (**E**) Risk of distant metastasis for different treatment groups. Significance was determined with hypothesis testing on risk ratio (**F**) Luminescence signal from freshly harvested mice lung at the endpoint (**G**) representative images of immunohistochemistry (IHC) performed on primary tumor probing for activated ERK1/2 (pERK1/2) from different treatment groups. (**H**) Luminescence signal intensity of primary tumor on day 13 by treatment. (**I**) Weight of freshly harvested tumor from differently treated groups at the endpoint. (**J**) H-score on pERK1/2 in each IHC sample is plotted by treatment. (**K**) Luminescence signal intensity from lung is plotted by treatment. *: *p* < 0.05, **: *p* < 0.005.

Next, we asked whether anti-IL-17RB response affects tumor growth and its subsequent dissemination. Aiming to provide an alternative to monoclonal antibody therapy, we believe the impact of the therapeutic use of the vaccine to be more clinically significant than the prophylactic use. Thus, we administered either the PBS, CRM197 or C:IRIS vaccine only after a successful tumor transplantation was confirmed by the in vivo imaging system (IVIS). Primary tumor growth and distance metastasis were monitored throughout the study (Figure 4A). We limited the tumor load to 1000 cells per mouse and adopted a rather radical vaccination scheme (Figure 4A). Using a parallel batch of mice (*n* = 5) vaccinated in the same way but without tumor transplantation, we observed that the total IgG titer reached dilution of over 20,000 fold (Figure 4B), suggesting that the intensive vaccination scheme enhanced immunogenicity more than less intensive schemes (Figure 3B). Around 20 days after transplantation, a considerable difference (*p* = 0.10) in primary tumor size was observed between PBS and C:IRIS vaccine treated groups (Figure 4D), roughly corresponding to the time when IgG response emerged (Figure 4B). This difference continued to increase and reached statistical significance (*p* < 0.05) at the end of the study (Figure 4D), which was also shown in the primary tumor weight at the endpoint (Figure 4I), suggesting that the IL-17RB specific IgG response suppressed primary tumor outgrowth. Moreover, the primary tumor from most C:IRIS recipients emitted far less luminescence comparing to control groups on day 13 (Figure 4C), and the intensity difference was significant (Figure 4H), suggesting that the primary tumor growth was inhibited by the C:IRIS vaccination. Altogether, these data proposed the therapeutic use of the C:IRIS vaccine in suppressing tumor growth to be highly promising.

In addition to primary tumor behaviors, we also investigated how therapeutic C:IRIS affects tumor migration. At the endpoint of the study, fresh lung tissue was harvested and briefly incubated in luciferin before being subjected to luminescence detection. We observed large, multi-focus metastatic colonies in control groups (PBS and Carrier) but only small, singular metastatic sites in the C:IRIS vaccine treated group (Figure 4F), indicating a lesser degree of disease progression and dissemination. This observation was further consolidated by quantitative analysis which showed significantly weaker luminescence intensity in the lung from the C:IRIS vaccine group as compared wit those from the control groups (Figure 4K), suggesting that the metastatic process has been delayed or suppressed. Furthermore, a significant drop in the distant metastasic risk (number of metastatic positive individuals divided by group size) was also found after C:IRIS vaccination using risk difference analysis (Figure 4E), further suggesting the effectiveness of C:IRIS being used in the therapeutic scenario. In short, therapeutic administration of C:IRIS can also be effective against cancer metastasis in the mouse model.

Previous research showed that IL-17RB contributes to tumorigenesis through ERK1/2 dependent cell activation [24], which, upon its activation, undergoes phosphorylation and relocates into the nucleus [32] (Figure 1B). We utilized immunohistochemistry to visualize and quantify activation of ERK1/2 (pERK1/2) in primary tumor sectioning. As expected, pERK1/2 was highly enriched in cell nuclei near the invasive edge of tumor samples where cells remained active and motile (Figure 4G), while counterparts from C:IRIS immunized primary tumors had significantly lower nuclear pERK1/2 (Figure 4G,J), suggesting that the inhibition of the ERK1/2 pathway in tumors from C:IRIS treated mice. This is consistent with our current understanding of IL-17RB and suggested that the C:IRIS vaccine could interrupt tumorigenesis through disruption of the IL-17RB signaling pathway in vivo.

## 4. Discussion

mAbs are among the most successful anticancer regiments [4,5,6]. However, they are not without their limitations [7,8]. Polyclonal antibodies (pAbs) produced by the host are functionally suitable as an alternative to mAbs, but their development as a cancer therapeutic is hindered by concerns about tumor-promoting B-cell responses [33,34]. This concept is gradually changing as accumulating evidence reveals that the mature B-cell response is actually an independent predictor of favorable prognosis in many types of cancer [35]. More recently, Wiedermann et al. developed a series of B-cell cancer vaccines directed against Her2 or PD-1 with great safety profiles and anti-cancer effects in the animal model [18,19], reiterating that, given a valid target, the active humoral response is suitable for a safe, economic and efficacious cancer therapy [11]. In this research, we developed a novel B-cell cancer vaccine by chemically conjugating the newly identified epitope from IL-17RB, an emerging cancer therapy target [36], and optimizing its bioactivity assisted by MALDI-TOF-MS. Finally, we validated its antitumor therapeutic effects in the animal study. 

To create an ideal B-cell vaccine, effective and immunogenic B-cell epitopes are of the greatest strategic importance. This can be identified by extensive or focused sequence screening [18,37], which is only suitable for linear epitope and potentially leads to unexhausted results. To address this concern, we designed our IRIS peptide in a structure-guided fashion referencing our previous study on IL-17RB [25], which fortuitously revealed IRIS epitope to be linear instead of conformational, meaning that IRIS peptides faithfully recapitulate native epitope upon synthesis and do not need further processing nor folding. Furthermore, both mouse and human IRIS homologs contain B-cell epitopes as predicted by the latest algorithm BepiPred2.0 [29] (Figure 1B). Thus, IRIS peptides are poised to direct the B-cell response against effective epitope on IL-17RB [24]. In order to enhance its immunogenicity, we designed an IRIS2 peptide (Table 1) which has doubled IRIS epitope load per peptide at the cost of creating non-specific epitope at the junction between two IRIS domains. Despite this, IRIS2 did outperform IRIS1 design in terms of bioactivity (Figure 3E), showing that the extra epitope effectively boosted immunogenicity. Whether fusing more epitopes per carrier protein generates a greater immune response is not investigated in this study but is worth inspecting in the future. Plus, multivalent peptide made of epitopes from different tumor-associated antigens is an intriguing option for future investigation.

In order to break immune tolerance towards IL-17RB, we conjugate IRIS peptides to CRM197, which has been successfully used in conjugate vaccines against bacterial capsular polysaccharides [17,38]. In recent years, research into peptide:CRM197 conjugates to tackle human diseases, ranging from Alzheimer’s to cancer, has become a vibrant and promising field [18,19,31]. Comparing to other popular candidates such as diphtheria toxoid (DT), tetanus toxoid (TT) and KLH, CRM197 is inherently non-toxic [39], highly immunogenic [40] and has low susceptibility [41], making CRM197 an ideal choice. Regarding its adverse effects (AEs), IL-17RB being expressed in normal tissues [36] raises concerns about inducing cell-mediated rejection type responses in these normal organs. Therefore, we chose alum as an adjuvant with the hope of inducing a pure humoral response instead of a cellular response to focus on signaling disruption and to minimize the cell-mediated cytotoxic effect [33,42]. We monitored each C:IRIS recipient for general conditions such as body weight, social behavior and skin lesions, none of which showed any C:IRIS specific AEs except for transient calcification at the injection site. We continued monitoring tumor-free C:IRIS recipients for over 100 days after the last injection without identifying any long term side effects. Therefore, our results suggested that C:IRIS is safe in vivo. Despite this, whether C:IRIS immunization interferes with normal IL-17 family functions like the host defense system [43] is still an open question and should be investigated in the future.

Chemical and biological characterization is of the utmost importance for drug development. For conjugate vaccines, hapten density estimation establishes consistency, enables optimization and is therefore crucial to a successful conjugate. While conjugates with more peptides are expected to induce stronger response [44], we observed excessive hapten density weakened the epitope affinity to antibodies (Figure 3A,B) and likely weakened its affinity to BCR as well, which may, in turn, alter B-cell activation capacity [45]. Thus, it is reasonable to assume an ideal range of hapten density for conjugate vaccines. To date, semi-quantitative techniques such as SDS-PAGE, biological activities and empirical predictions remain the mainstay way to determine hapten density for peptide vaccines [18,19,31,37,46], none of which renders information detailed enough for effective quality control. We reason that peptide-protein conjugation creates a mixture of contents with varying activation numbers, peptide loads and positions and are too heterogeneous to be distinctively separated by SDS-PAGE or SEC (Figure 2B,C). In 2018, Jaffe et al. overcame this limitation with electrospray ionization time-of-flight mass spectrometry (ESI-TOF-MS) [47], however, for longer peptides like IRIS2, mass spectrum of multiply charged samples generated by ESI extensively overlapped and was uninterpretable. We therefore turned to MALDI-TOF-MS, which generates mainly singly or low charged ions, and applied mass spectrum deconvolution to retrieve a relative abundance of different hapten densities (Figure 2D). Several factors were considered during the quantification analysis using MALDI-TOF-MS, including the ionization/desorption, ion transmission and detection efficiencies [48]. First, individual C:IRIS vaccines consisted of different peptide loads of species, which were mixed homogenously in solution. C:IRIS from different peptide loads should have similar chemical properties and ionization efficiencies. They were prepared onto the MALDI sample plate, desorbed, ionized and accelerated under the exact same conditions. Second, a time-of-flight mass analyzer has high ion transmission, especially the short flight path of the instrument in this research, resulting in low ion loss. Finally, the sensitivity of ion detectors for within a small mass region was roughly constant, making quantitative signal comparison possible. This enabled reliable and reproducible hapten density estimation for the C:IRIS vaccine and may also benefit the development of other potential peptide-protein conjugates. 

Clinically, vaccines have the option to be administered prophylactically. However, the concept of breaking tolerance against self-epitope in a healthy person is somewhat alarming. To date, none of the five cancer vaccines approved by FDA for prophylactic use target self-antigen [13], strongly suggesting that the therapeutical use of the C:IRIS vaccine may have greater clinical impacts. Therefore, we demonstrated that the therapeutic use of C:IRIS, which showed great anti-tumor effects through disruption of ERK1/2 activation, moves the critical signal transduction event downstream to IL-17RB [24]. Surprisingly, we observed a general trend for suppressed disease progression after the therapeutic CRM197 injection (Figure 4D,E,H–K), indicating that CRM197 has an intrinsic anti-tumor property. This is consistent with other groups’ results that therapeutically administered CRM197, which suppressed tumor growth in a different animal model [49]. In this sense, the C:IRIS vaccine itself can be considered a combination therapy. Still, we envision the C:IRIS cancer vaccine to be combined with other major anti-cancer regiments such as surgical resection, radio therapy and chemotherapy to benefit patients.

## 5. Conclusions

In summary, we designed a peptide conjugate vaccine targeting interleukin 17 receptor B which generated potent and specific polyclonal antibodies that were able to replicate therapeutic effects like its mAb counterpart, validating the concept that of a B-cell cancer vaccine as an efficacious and economic alternative to mAbs in cancer therapy. We also standardized the characterization method for conjugate vaccines with large peptides that are not suitable to be analyzed by ESI-TOF-MS, enabling immunogenicity optimization of conjugate vaccines to achieve anti-tumor effect in a pre-clinical model. Our work paves the way for a safe and cost-effective anti-cancer therapy and may benefit future B-cell cancer vaccine development and translation.

## Figures and Tables

**Table 1:** IL-17RB inactivation site (IRIS) homologue sequence and synthesized peptide design.

ID.	Name	Sequence	Description
1	bIRIS	Val_19_ Arg His Thr Leu Thr_24_ Pro Gly Asp Leu Arg_29_ Asp Leu	Bovine IRIS
2	mIRIS	Val_19_ Gln His Thr Leu Thr_24_ Pro Gly Asp Leu Arg_29_ Asp Leu	IRIS homologous in Mus musculus
3	hIRIS	Leu_19_ Gln His Asp Leu Ile_24_ Pro Gly Asp Leu Arg_29_ Asp Leu	IRIS homologous in Homo sapiens
4	mIRIS1	Ac (Val_19_ Gln His Thr Leu Thr_24_ Pro Gly Asp Leu Arg_29_ Asp Leu Gly Gly)_1_ Cys	Synthesised peptide with 1 Mus musculus IRIS
5	hIRIS1	Ac (Leu_19_ Gln His Asp Leu Ile_24_ Pro Gly Asp Leu Arg_29_ Asp Leu Gly Gly)_1_ Cys	Synthesised peptide with 1 Homo sapiens IRIS
6	mIRIS2	Ac (Val_19_ Gln His Thr Leu Thr_24_ Pro Gly Asp Leu Arg_29_ Asp Leu Gly Gly)_2_ Cys	Synthesised peptide with 2 Mus musculus IRIS
7	hIRIS2	Ac (Leu_19_ Gln His Asp Leu Ile_24_ Pro Gly Asp Leu Arg_29_ Asp Leu Gly Gly)_2_ Cys	Synthesised peptide with 2 Homo sapiens IRIS

## Data Availability

Thw B-cell epitope prediction algorithm is available from IEDB Analysis Resource (accessed on 18 January 2021, http://tools.iedb.org/bcell/). Protein sequence information is available on UniProt (accessed on 18 January 2021, https://www.uniprot.org/). Data presented in this study is available in the manuscript.

## References

[1] Slamon D.J., Clark G.M., Wong S.G., Levin W.J., Ullrich A., McGuire W.L. (1987). Human breast cancer: Correlation of relapse and survival with amplification of the HER-2/neu oncogene. Science.

[2] Salomon D.S., Brandt R., Ciardiello F., Normanno N. (1995). Epidermal growth factor-related peptides and their receptors in human malignancies. Crit. Rev. Oncol..

[3] Piccart-Gebhart M.J., Procter M., Leyland-Jones B., Goldhirsch A., Untch M., Smith I., Gianni L., Baselga J., Bell R.H., Jackisch C. (2005). Trastuzumab after Adjuvant Chemotherapy in HER2-Positive Breast Cancer. N. Engl. J. Med..

[4] Sharma P., Allison J.P. (2015). The future of immune checkpoint therapy. Science.

[5] Borghaei H., Paz-Ares L., Horn L., Spigel D.R., Steins M., Ready N.E., Chow L.Q., Vokes E.E., Felip E., Holgado E. (2015). Nivolumab versus Docetaxel in Advanced Nonsquamous Non–Small-Cell Lung Cancer. N. Engl. J. Med..

[6] Reck M., Rodríguez-Abreu D., Robinson A.G., Hui R., Csőszi T., Fülöp A., Gottfried M., Peled N., Tafreshi A., Cuffe S. (2016). Pembrolizumab versus Chemotherapy for PD-L1–Positive Non–Small-Cell Lung Cancer. N. Engl. J. Med..

[7] Kaumaya P.T. (2020). B-cell epitope peptide cancer vaccines: A new paradigm for combination immunotherapies with novel checkpoint peptide vaccine. Future Oncol..

[8] Hernandez I., Bott S.W., Patel A.S., Wolf C.G., Hospodar A.R., Sampathkumar S., Shrank W.H. (2018). Pricing of Monoclonal Antibody Therapies: Higher If Used for Cancer?. Am. J. Manag. Care.

[9] Lambert A.W., Pattabiraman D.R., Weinberg R.A. (2017). Emerging Biological Principles of Metastasis. Cell.

[10] Riedel S. (2005). Edward Jenner and the history of smallpox and vaccination. Proc. Bayl. Univ. Med. Cent..

[11] Kametani Y., Miyamoto A., Tsuda B., Tokuda Y. (2015). B Cell Epitope-Based Vaccination Therapy. Antibodies.

[12] Malonis R.J., Lai J.R., Vergnolle O. (2020). Peptide-Based Vaccines: Current Progress and Future Challenges. Chem. Rev..

[13] Crews D.W., Dombroski J.A., King M.R. (2021). Prophylactic Cancer Vaccines Engineered to Elicit Specific Adaptive Immune Response. Front. Oncol..

[14] Hollingsworth R.E., Jansen K. (2019). Turning the corner on therapeutic cancer vaccines. Npj Vaccines.

[15] Toes R.E., Offringa R., Blom R.J., Melief C.J., Kast W.M. (1996). Peptide vaccination can lead to enhanced tumor growth through specific T-cell tolerance induction. Proc. Natl. Acad. Sci. USA.

[16] Toes R.E., Blom R.J., Offringa R., Kast W.M., Melief C.J. (1996). Enhanced tumor outgrowth after peptide vaccination. Functional deletion of tumor-specific CTL induced by peptide vaccination can lead to the inability to reject tumors. J. Immunol..

[17] Pichichero M.E. (2013). Protein carriers of conjugate vaccines: Characteristics, development, and clinical trials. Hum. Vaccines Immunother..

[18] Tobias J., Battin C., Linhares A.D.S., Lebens M., Baier K., Ambroz K., Drinić M., Högler S., Inic-Kanada A., Garner-Spitzer E. (2020). A New Strategy Toward B Cell-Based Cancer Vaccines by Active Immunization With Mimotopes of Immune Checkpoint Inhibitors. Front. Immunol..

[19] Tobias J., Jasinska J., Baier K., Kundi M., Ede N., Zielinski C., Wiedermann U. (2017). Enhanced and long term immunogenicity of a Her-2/neu multi-epitope vaccine conjugated to the carrier CRM197 in conjunction with the adjuvant Montanide. BMC Cancer.

[20] Dakappagari N.K., Pyles J., Parihar R., Carson W.E., Young D.C., Kaumaya P.T.P. (2003). A Chimeric Multi-Human Epidermal Growth Factor Receptor-2 B Cell Epitope Peptide Vaccine Mediates Superior Antitumor Responses. J. Immunol..

[21] Allen S.D., Garrett J.T., Rawale S.V., Jones A.L., Phillips G., Forni G., Morris J.C., Oshima R.G., Kaumaya P.T.P. (2007). Peptide Vaccines of the HER-2/neu Dimerization Loop Are Effective in Inhibiting Mammary Tumor Growth In Vivo. J. Immunol..

[22] Huang C.-K., Yang C.-Y., Jeng Y.-M., Chen C.-L., Wu H.-H., Chang Y.-C., Ma C., Kuo W.-H., Chang K.-J., Shew J.-Y. (2014). Autocrine/paracrine mechanism of interleukin-17B receptor promotes breast tumorigenesis through NF-κB-mediated antiapoptotic pathway. Oncogene.

[23] Huang S., Wei P., Hwang-Verslues W.W., Kuo W., Jeng Y., Hu C., Shew J., Huang C.-S., Chang K., Lee E.Y. (2017). TGF-β1 secreted by Tregs in lymph nodes promotes breast cancer malignancy via up-regulation of IL-17RB. MBO Mol. Med..

[24] Wu H.-H., Hwang-Verslues W.W., Lee W.-H., Huang C.-K., Wei P.-C., Chen C.-L., Shew J.-Y., Lee E.Y.-H., Jeng Y.-M., Tien Y.-W. (2015). Targeting IL-17B–IL-17RB signaling with an anti–IL-17RB antibody blocks pancreatic cancer metastasis by silencing multiple chemokines. J. Exp. Med..

[25] Lee W.-H., Chen X., Liu I.-J., Lee J.-H., Wu H.-C., Hu C.-M., Wang S.-K., Lee W.-H., Ma C. (2022). Crystal structure of Interleukin-17B receptor in complex with a mouse neutralizing monoclonal antibody D9 for guiding humanization and affinity maturation. Nat. Commun..

[26] Cai Y.-H., Lai Y.-H., Wang Y.-S. (2015). Coupled Space- and Velocity-Focusing in Time-of-Flight Mass Spectrometry—A Comprehensive Theoretical Investigation. J. Am. Soc. Mass Spectrom..

[27] Gibb S., Strimmer K. (2012). MALDIquant: A versatile R package for the analysis of mass spectrometry data. Bioinformatics.

[28] Werle M., Bernkop-Schnürch A. (2006). Strategies to improve plasma half life time of peptide and protein drugs. Amino Acids.

[29] Jespersen M.C., Peters B., Nielsen M., Marcatili P. (2017). BepiPred-2.0: Improving sequence-based B-cell epitope prediction using conformational epitopes. Nucleic Acids Res..

[30] Harrisson S. (2018). The downside of dispersity: Why the standard deviation is a better measure of dispersion in precision polymerization. Polym. Chem..

[31] Vingtdeux V., Zhao H., Chandakkar P., Acker C.M., Davies P., Marambaud P. (2016). A modification-specific peptide-based immunization approach using CRM197 carrier protein: Development of a selective vaccine against pyroglutamate Aβ peptides. Mol. Med..

[32] Plotnikov A.N., Flores K., Maik-Rachline G., Zehorai E., Kapri-Pardes E., Berti D.A., Hanoch T., Besser M.J., Seger R. (2015). The nuclear translocation of ERK1/2 as an anticancer target. Nat. Commun..

[33] Vermaelen K. (2019). Vaccine Strategies to Improve Anti-cancer Cellular Immune Responses. Front. Immunol..

[34] Disis M.L. (2010). Immune Regulation of Cancer. J. Clin. Oncol..

[35] Fridman W.H., Petitprez F., Meylan M., Chen T.W.-W., Sun C.-M., Roumenina L.T., Sautès-Fridman C. (2021). B cells and cancer: To B or not to B?. J. Exp. Med..

[36] Bastid J., Dejou C., Docquier A., Bonnefoy N. (2020). The Emerging Role of the IL-17B/IL-17RB Pathway in Cancer. Front. Immunol..

[37] Zhao Q., Gao Y., Xiao M., Huang X., Wu X. (2021). Synthesis and immunological evaluation of synthetic peptide based anti-SARS-CoV-2 vaccine candidates. Chem. Commun..

[38] Bröker M., Costantino P., DeTora L., McIntosh E.D., Rappuoli R. (2011). Biochemical and biological characteristics of cross-reacting material 197 (CRM197), a non-toxic mutant of diphtheria toxin: Use as a conjugation protein in vaccines and other potential clinical applications. Biologicals.

[39] Malito E., Bursulaya B., Chen C., Surdo P.L., Picchianti M., Balducci E., Biancucci M., Brock A., Berti F., Bottomley M.J. (2012). Structural basis for lack of toxicity of the diphtheria toxin mutant CRM197. Proc. Natl. Acad. Sci. USA.

[40] Prasad A.K., Kim J.-H., Gu J. (2018). Design and Development of Glycoconjugate Vaccines. Am. Chem. Soc..

[41] Tontini M., Berti F., Romano M., Proietti D., Zambonelli C., Bottomley M., De Gregorio E., Del Giudice G., Rappuoli R., Costantino P. (2013). Comparison of CRM197, diphtheria toxoid and tetanus toxoid as protein carriers for meningococcal glycoconjugate vaccines. Vaccine.

[42] Wisniewski T., Goñi F. (2015). Immunotherapeutic approaches for Alzheimer’s disease. Neuron.

[43] Gu C., Wu L., Li X. (2013). IL-17 family: Cytokines, receptors and signaling. Cytokine.

[44] Li Q., Rodriguez L.G., Farnsworth D.F., Gildersleeve J.C. (2010). Effects of Hapten Density on the Induced Antibody Repertoire. ChemBioChem.

[45] Sáez de Guinoa J., Barrio L., Mellado M., Carrasco Y.R. (2011). CXCL13/CXCR5 signaling enhances BCR-triggered B-cell activation by shaping cell dynamics. Blood.

[46] Abbas I.M., Schwaar T., Bienwald F., Weller M.G. (2017). Predictable Peptide Conjugation Ratios by Activation of Proteins with Succinimidyl Iodoacetate (SIA). Methods Protoc..

[47] Jaffe J., Wucherer K., Sperry J., Zou Q., Chang Q., Massa M.A., Bhattacharya K., Kumar S., Caparon M., Stead D. (2018). Effects of Conformational Changes in Peptide–CRM197 Conjugate Vaccines. Bioconjugate Chem..

[48] Wang C.-C., Lai Y.-H., Ou Y.-M., Chang H.-T., Wang Y.-S. (2016). Critical factors determining the quantification capability of matrix-assisted laser desorption/ionization– time-of-flight mass spectrometry. Philos. Trans. R. Soc. A Math. Phys. Eng. Sci..

[49] Kunami N., Yotsumoto F., Ishitsuka K., Fukami T., Odawara T., Manabe S., Ishikawa T., Tamura K., Kuroki M., Miyamoto S. (2011). Antitumor Effects of CRM197, A Specific Inhibitor of HB-EGF, in T-Cell Acute Lymphoblastic Leukemia. Anticancer Res..

